# Lymphomas of the submandibular gland: a nationwide cohort study

**DOI:** 10.1007/s00405-024-09008-x

**Published:** 2024-10-08

**Authors:** Fahd Al-Shahrestani, Ahmed Ehsan Al-Khafaf, Zain Asheer, Jelena Jelicic, Iman Chanchiri, Catharina E. Blocher, Anne Kathrine Aalling Sørensen, Lars Møller Pedersen, Lise Mette Rahbek Gjerdrum, Steffen Heegaard, Preben Homøe

**Affiliations:** 1 Department of Otorhinolaryngology and Maxillofacial Surgery, Zealand University Hospital, Køge, Denmark; 2 Department of Clinical Medicine, University of Copenhagen, Copenhagen, Denmark; 3 Department of Ophthalmology and Pathology, Rigshospitalet, Copenhagen, Denmark; 4 Department of Pathology, Zealand University Hospital, Roskilde, Denmark; 5 Department of Hematology, Zealand University Hospital, Roskilde, Denmark; 6 Department of Hematology, Odense University Hospital, Odense, Denmark; 7 Department of Hematology, Vejle Hospital, Sygehus Lillebaelt, Vejle, Denmark; 8 Department of Hematology, Gødstrup Hospital, Herning, Denmark; 9 Department of Hematology, Aalborg University Hospital, Aalborg, Denmark

**Keywords:** Submandibular gland, Autoimmune disease, Survival, Lymphoma

## Abstract

**Objective:**

This study explores the epidemiology, incidence, and survival outcomes associated with lymphomas of the submandibular gland (SMG) and examines the influence of autoimmune diseases on these parameters.

**Methods:**

This retrospective nationwide cohort study analysed data from patients diagnosed with SMG lymphomas in Denmark between 2000 and 2020. Information was extracted from medical records, the National Pathology Register, and the Danish Lymphoma Database. Survival analyses were conducted using Kaplan-Meier curves, log-rank tests, and Cox proportional hazards models, focusing on lymphoma subtypes and autoimmune diseases.

**Results:**

The cohort consisted of 101 patients with a lymphoma diagnosis and involvement of the SMG. Large B-cell lymphoma (LBCL) was diagnosed in 33 cases (32.7%), follicular lymphoma (FL) in 29 cases (28.7%), extranodal marginal zone lymphoma (EMZL) in 27 cases (26.7%), and 12 cases (11.9%) with other subtypes. EMZL had a significantly longer overall survival (OS) compared to other subtypes, with a median OS of 12.4 years (95% CI 11.2–12.4) vs. 8.4 years (95% CI 6.0-12.2). EMZL and FL showed favourable 5-year OS rates of 95% and 89%, respectively. LBCL had a 5-year OS rate of 65%. Age over 60 significantly negatively impacted OS. Traditional poor prognostic indicators did not significantly affect OS. A notable association between EMZL and autoimmune diseases was observed, particularly with Sjögren’s syndrome, indicated by an increased relative risk of 2.67 (CI 95% 0.45–16.01).

**Conclusions:**

Lymphomas of the SMG are rare and have ambiguous clinical presentations. This study provides novel epidemiological, clinical, and prognostic information.

## Introduction

Lymphomas represent the second most common cancer type in the head and neck region, notably affecting the salivary glands including the submandibular gland [1, 2]. Submandibular gland lymphomas present challenges in diagnosis and management due to its rarity and diverse manifestations [1, 3, 4]. Malignant tumours of the submandibular gland often present as painless swellings and may not be diagnosed until they become symptomatic or are incidentally discovered during imaging for unrelated issues, complicating their treatment [5]. The literature reveals limited information on submandibular gland lymphomas, with existing studies often being small, outdated single-centre studies or based on databases of varying quality [1, 3, 4]. This limitation affects the value of results from previous studies, especially considering the ongoing changes and developments in classifications and treatments [6]. 

The incidence of autoimmune diseases including Sjögren’s syndrome (SS), systemic lupus erythematosus (SLE), and rheumatoid arthritis (RA) has progressively increased in recent years.^4^ Autoimmune diseases have been linked to a heightened risk of developing non-Hodgkins lymphoma (NHL) due to continuous antigenic stimulation,^5–7^ which may cause molecular and genetic alterations leading to unregulated B cell proliferation and malignant transformation.^8–10^ Nonetheless, the lymphomagenesis associated with autoimmune diseases remains not fully elucidated.^9,10^

This study provides a comprehensive national epidemiological and clinical overview of lymphomas in the submandibular gland in a nation-wide population-based study. The primary aims of the study were to provide data on lymphoma subtypes including incidence and survival rates of lymphoma involving the submandibular gland and to investigate their relationship to autoimmune diseases.

## Materials and methods

### Study design and data collection

The study is a retrospective observational cohort study. The cohort comprises all patients diagnosed with submandibular gland lymphoma in Denmark between 2000 and 2020.

Data was collected from medical records, tissue samples, the National Pathology Register (NPR), the Danish lymphoma database (LyFo), the National Patient Register (LPR), and the Cause of Death Register (DAR). Medical records and LyFo supplied the following data: lymphoma subtype, anatomical site, laterality, bone marrow and lymph node involvement, date of lymphoma diagnosis, presenting symptom for lymphomas, laboratory test results, concomitant autoimmune disease (SS, RA and SLE), imaging modality, tumour size, Ann Arbor stage, treatment, response to treatment, response criteria [7], age, sex, date of death, and cause of death. If data were available from multiple sources, cross-verification was performed as validation.

Patients with a lymphoma diagnosis and salivary gland involvement were identified through the LyFo registry. The LyFo registry has a 95% coverage of all Danish lymphoma patients, along with high data quality and completeness, according to a previously reported quality assessment [8]. LyFo does not differentiate between the different salivary glands, and NPR or medical records were used to determine the anatomical site of involvement. NPR reports were used to cross-validate the Systematized Nomenclature of Medicine (SNOMED) codes and to exclude non-salivary gland materials, such as extraglandular lymph nodes. All included cases were embedded in submandibular gland tissue. Unlike the parotid gland, the submandibular gland does not contain lymph nodes. Therefore, parotid gland lymphomas were not included in this study.

Following identification of the cohort, patients were divided into two groups: primary and secondary lymphomas. Primary lymphoma was defined as involvement of the submandibular gland at the time of a biopsy-verified lymphoma without a prior lymphoma diagnosis. Secondary lymphoma was defined as involvement of the submandibular gland at the time of a biopsy-verified lymphoma with a prior lymphoma diagnosis at least three months apart.

### Lymphoma classification

All cases were reviewed by expert hematopathologist in accordance with the latest WHO classification [6]. Following review of the pathology reports, selected cases were re-evaluated to ensure diagnostic accuracy.

### Immunohistochemistry and FISH

In large B-cell lymphoma (LBCL) and extranodal marginal zone lymphoma (EMZL) cases, immunohistochemical evaluation and fluorescence in situ hybridization (FISH) were performed. The following panel of antibodies was used for validation: anti-CD3 (poly, AKO), CD5 (4C7, Agilent), CD10 (SP67, DAKO), CD20 (L26, DAKO), CD23 (DAK-CD23, DAKO), CD30 (Ber-H2, Agilent), CD79a (JCB117, Agilent), cyclin D1 (EP12, DAKO), PAX5 (DAK-PAX2, DAKO), cMYC (EP121, DAKO), BCL2 (124, Agilent), BCL6 (PG-B6p, DAKO), MUM-1 (MUM1p, DAKO), and κ (A8B5, Agilent) and λ (HP6054, Agilent) light chains [9]. 

### DNA extraction and BIOMED 2 clonality testing for Ig

For DNA extraction and BIOMED-2 clonality testing for Ig, tissue sections were deparaffinized and rehydrated using standard xylene and ethanol washes. DNA was extracted using the QIAamp DNA Mini Kit (Qiagen) according to the manufacturer’s instructions. Clonality testing was performed using the BIOMED-2 multiplex PCR protocol targeting IGH, IGK, and IGL gene rearrangements. PCR products were analysed by capillary electrophoresis (ABI 3130 Genetic Analyzer), and data were interpreted using GeneMapper software. A monoclonal pattern indicated a clonal B-cell population consistent with a lymphoma, whereas a polyclonal pattern suggested a reactive process [9]. 

### Statistics

Continuous variables were presented as median values (range or 95% CI), while frequencies were used for categorical variables. Logistic regression analyses were employed to determine the relative risk (RR) with 95% confidence intervals based on exposure to autoimmune disease or not. Overall survival (OS) was defined as the time from the lymphoma diagnosis to death from any cause or last follow-up. Time to progression (TTP) was recorded as the time between lymphoma and documented lymphoma progression or death as a result of the lymphoma. Time-to-event analyses were plotted using the Kaplan-Meier method and compared with the log rank test. To assess the concurrent influence of several factors on survival, a Cox proportional hazards model was applied. Stata Statistical Software version 18.0 (College Station, TX: StataCorp LLC) was used for the calculations. The incidence was calculated using the average number of cases per year from 2000 to 2020 and the average population figures from Statistics Denmark for the same period [10]. The proportion of salivary gland lymphomas was determined based on the average number of NHL cases in Denmark, with data available only from 2010 to 2020 [11]. 

## Results

### Study population

**Fig. 1:**
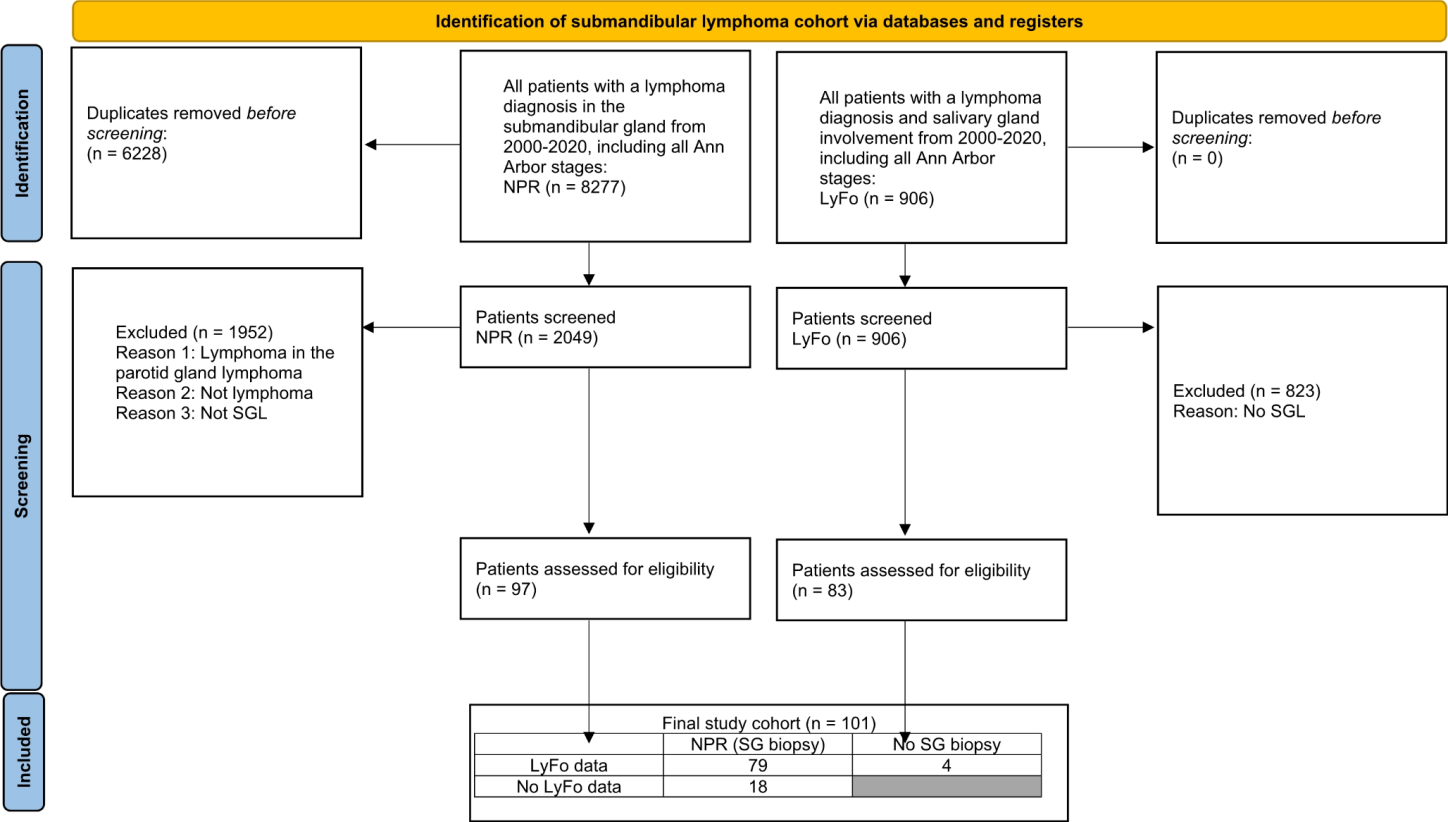
Flowchart depicting the patient selection process for inclusion in the study cohort from initial identification in databases and registries through screening and assessment for eligibility, based on lymphoma diagnosis in the submandibular gland between 2000–2020. NPR = National Pathology Register, LyFo = Danish lymphoma database, SG = salivary gland, SGL = salivary gland lymphoma

The final study cohort of lymphoma in the submandibular gland consisted of 101 patients with 44 males (43.6%) and 57 females (56.4%), and a median age at presentation of 67 years (range 36–99 years) (Table 1). Flowchart of patient selection is shown in Fig. 1. The majority of the cases were primary lymphomas (*n* = 81, 80.2%) (Table 1). The nationwide incidence of lymphoma in the submandibular gland in Denmark was 5/year/5,549,211 people (0.09/ 100,000 person-years) [10]. Based on the average of 1125 new cases of NHL from 2010 to 2020 in Denmark, submandibular gland lymphomas constituted 0.45% of all NHLs [11]. 

**Table 1:** Demographic and clinical characteristics of patients with Submandibular Gland Lymphoma

			Subtypes			
		**All**	**EMZL**	**LBCL**	**FL**	**Other NHL**
Total	**(%)**	101	27 (26.7)	33 (32.7)	29 (28.7)	12 (11.9)
Sex	**Male**	44 (43.6)	10 (37.0)	16 (48.5)	10 (34.5)	8 (66.7)
	**Female**	57 (56.4)	17 (63.0)	17 (51.5)	19 (65.5)	4 (33.3)
Age at diagnosis	**Median (Range) years**	67 (36–99)	59 (36–85)	71 (41–99)	68 (49–86)	65 (51–71)
Disease stage						
	**Primary lymphoma**	81 (80.2)	23 (85.2)	29 (87.9)	20 (69.0)	9 (75.0)
	**Secondary lymphoma**	20 (19.8)	4 (14.8)	4 (12.1)	9 (31.0)	3 (25.0)
	**NA**	-	-	-	-	-
Laterality						
	**Unilateral**	72 (96.0)	20 (95.2)	27 (93.1)	20 (100.0)	5 (55.6)
	**Bilateral**	3 (4.0)	1 (4.8)	2 (6.9)	-	-
	**NA**	6	2	-	-	4
Duration of symptoms (months)	**Median (range)**	1 (0–120)	2 (0–120)	1 (0–75)	1 (0–12)	0 (0–6)
	**NA**	4	-	-	-	4
Debut symptom						
	**None**	14 (18.2)	6 (26.1)	4 (13.8)	3 (15.0)	1 (20.0)
	**Palpable tumor**	52 (67.5)	13 (56.5)	21 (72.4)	16 (80.0)	2 (40.0)
	**Tumor in another location in the head and neck**	8 (10.4)	3 (13.0)	3 (10.3)	1 (5.0)	1 (20.0)
	**Other symptoms (nerve invasion**,** trismus**,** xerostomia**,** pain)**	3 (3.9)	1 (4.3)	1 (3.4)	-	1 (20.0)
	**NA**	4	-	-	-	4
B-symptoms						
	**No**	65 (85.5)	19 (86.4)	21 (77.8)	16 (88.9)	9 (100.0)
	**Yes**	11 (14.5)	3 (13.6)	6 (22.2)	2 (11.1)	-
	**NA**	5	1	2	2	
Ann Arbor stage						
	**IE**	28 (38.4)	15 (68.2)	8 (29.6)	5 (33.3)	-
	**IIE**	11 (15.1)	1 (4.5)	7 (25.9)	2 (13.3)	1 (11.1)
	**III**	15 (20.5)	1 (4.5)	7 (25.9)	4 (26.7)	3 (33.3)
	**IV**	19 (26.0)	5 (22.7)	5 (18.5)	4 (26.7)	5 (55.6)
	**NA**	8	1	2	5	-*
Bone marrow involvement						
	**No**	62 (76.5)	18 (78.3)	24 (82.8)	16 (80.0)	4 (44.4)
	**Yes**	19 (23.5)	5 (21.7)	5 (17.2)	4 (20.0)	5 (55.6)
	**NA**	-	-	-	-	-
Elevated LDH						
	**No**	55 (78.6)	16 (76.2)	21 (80.8)	11 (78.6)	7 (77.8)
	**Yes**	15 (21.4)	5 (23.8)	5 (19.2)	3 (21.4)	2 (22.2)
	**NA**	11	2	3	6	-
Submandibular tumor size						
	**0–2 cm**	19 (30.2)	6 (37.5)	6 (24.0)	5 (29.4)	2 (40.0)
	**2**,**01–5 cm**	34 (54.0)	9 (56.3)	14 (56.0)	9 (52.9)	2 (40.0)
	**5**,**01–10 cm**	10 (15.9)	1 (6.3)	5 (20.0)	3 (17.6)	1 (20.0)
	**> 10 cm**	-	-	-	-	-
	**NA**	18	7	4	3	4

EMZL: Extra nodal marginal zone lymphoma, LBCL: large B-cell lymphoma, FL: follicular lymphoma, NHL: Non-hodgkin lymphoma, other NHL: small lymphocytic lymphoma, Mantle cell lymphoma, peripheral T-cell lymphoma

Unilateral involvement was predominant (*n* = 72, 96%). Duration of symptoms before diagnosis varied, with a median duration of 1 month (range 0-120 months). The symptoms were primarily reported as a tumour in the submandibular region (*n* = 52, 67.5%). Other initial presentations, such as xerostomia, pain or affected hypoglossal or lingual nerve function, were less commonly noted. B-symptoms were only rarely reported (*n* = 11, 14.5%).

Of the lymphoma subtypes, LBCL was the most frequently diagnosed (*n* = 33, 32.7%), followed by follicular lymphoma (FL) (*n* = 29, 28.7%) and EMZL (*n* = 27, 26.7%). Other subtypes mainly included mantle cell lymphoma (MCL), and T-cell lymphoma were less frequent. Hodgkin lymphoma was not found in any cases.

According to Ann Arbor staging with Lugano criteria, the largest group of patients was identified with stage IE (*n* = 28, 38.4%). Bone marrow involvement at the time of presentation was positive in 19 patients (23.5%) and elevated lactate dehydrogenase (LDH) levels were found in 14 patients (21.4%). The most common tumour size at presentation was between 2 and 5 cm (*n* = 34, 54%).

Median time from diagnosis to initiation of treatment of primary lymphoma or decision was 29 days. Treatment modalities ranged from watch and wait to surgery, with patients receiving one or more types of treatment simultaneously. The most common treatment modalities were chemotherapy, radiotherapy and Rituximab (Table 2). Other treatments, e.g. glucocorticoids or allogenic stem-cell transplantation, were only noted in 4 patients (3.9%).

**Table 2:** Treatment and outcomes in Submandibular Gland Lymphoma

			Subtyper			
		**All**	**EMZL**	**DLBCL**	**FL**	**Others**
Total	**(%)**	81	23 (28.40)	29 (35.80)	20 (24.69)	4 (4.94)
TIME TO treatment/decision, days	**Median**	29	46	25.5	36	19
treatment of primary lymphoma						
	**Watch and wait**	21 (20.8)	6 (22.2)	6 (18.2)	8 (27.6)	1 (8.3)
	**Rituximab**	36 (35.6)	3 (11.1)	21 (63.6)	9 (31.0)	3 (25.0)
	**Chemotherapy**	43 (42.8)	3 (11.1)	25 (75.8)	11 (37.9)	4 (33.3)
	**Radiotherapy**	44 (43.6)	16 (59.3)	12 (36.4)	12 (41.4)	4 (33.3)
	**Surgery**	20 (19.8)	6 (22.2)	5 (15.2)	3 (10.3)	6 (50.0)
	**Other treatment**	20 (19.8)	6 (22.2)	5 (15.2)	3 (10.3)	6 (50.0)
Response after first line treatment (Lugano criteria)						
	**Complete response**	50	14 (60.9)	20 (69.0)	11 (55.0)	5 (55.6)
	**Partial response**	4	1 (4.3)	1 (3.4)	1 (5.0)	1 (11.1)
	**Stable disease**	4	2 (8.7)	-	2 (10.0)	-
	**Progressive Disease**	10	1 (4.3)	3 (10.3)	3 (15.0)	3 (33.3)
	**Not Evaluated**	2	1 (4.3)	1 (3.4)	-	-
	**No response evaluation**	-	-	-	-	-
	**Not treated**	4	2 (8.7)	1 (3.4)	1 (5.0)	-

### Features of large cell B-cell lymphomas in the submandibular gland

**Fig. 2:**
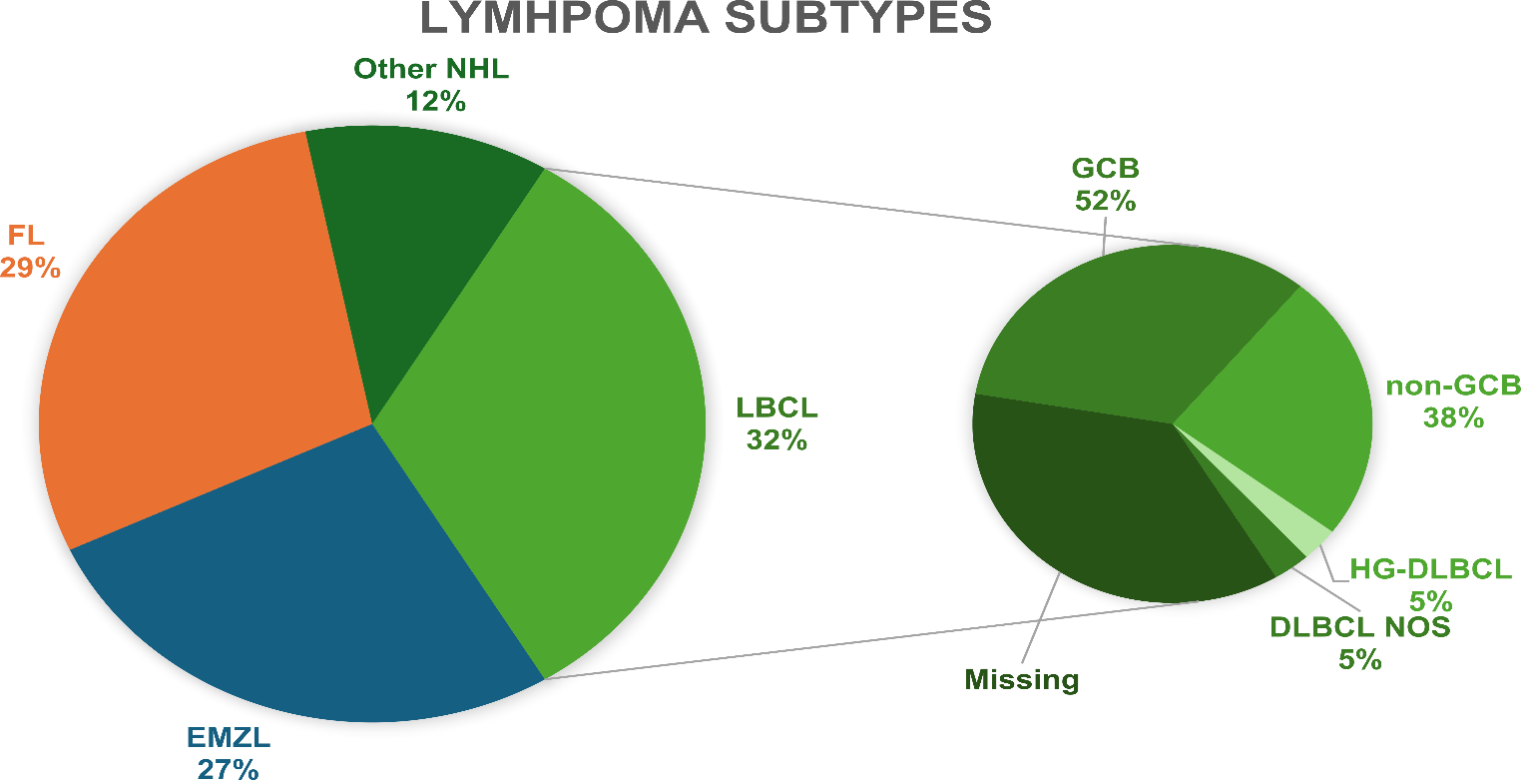
Pie-chart distribution of the lymphoma subtypes in the submandibular gland, including LBCL subtypes. FL: Follicular Lymphoma, EMZL: Extranodal Marginal Zone Lymphoma, LBCL: DLBCL: Diffuse Large B-Cell Lymphoma, HG-LBCL: High Grade DLBCL, GCB: Germinal Centre B-cell like, NOS: Not Otherwise Specified

In our study, we identified two subtypes of LBCL: diffuse large B-cell lymphoma (DLBCL) (*n* = 19, 90%) and high-grade B-cell lymphoma (HGBL) (*n* = 1, 5%) with MYC and BCL2 rearrangements. The majority of DLBCL cases were Germinal Centre B-cell-like (GCB) (*n* = 11, 52%). An additional category, DLBCL not otherwise specified (*n* = 1, 5%), was due to a poorly fixed FFPE sample that produced unusable results in FISH testing. We were able to retrieve 21 tissue samples, with the remaining 12 samples classified as missing (Fig. 2). Common characteristics at presentation can be seen in Table 1.

**Fig. 3:**
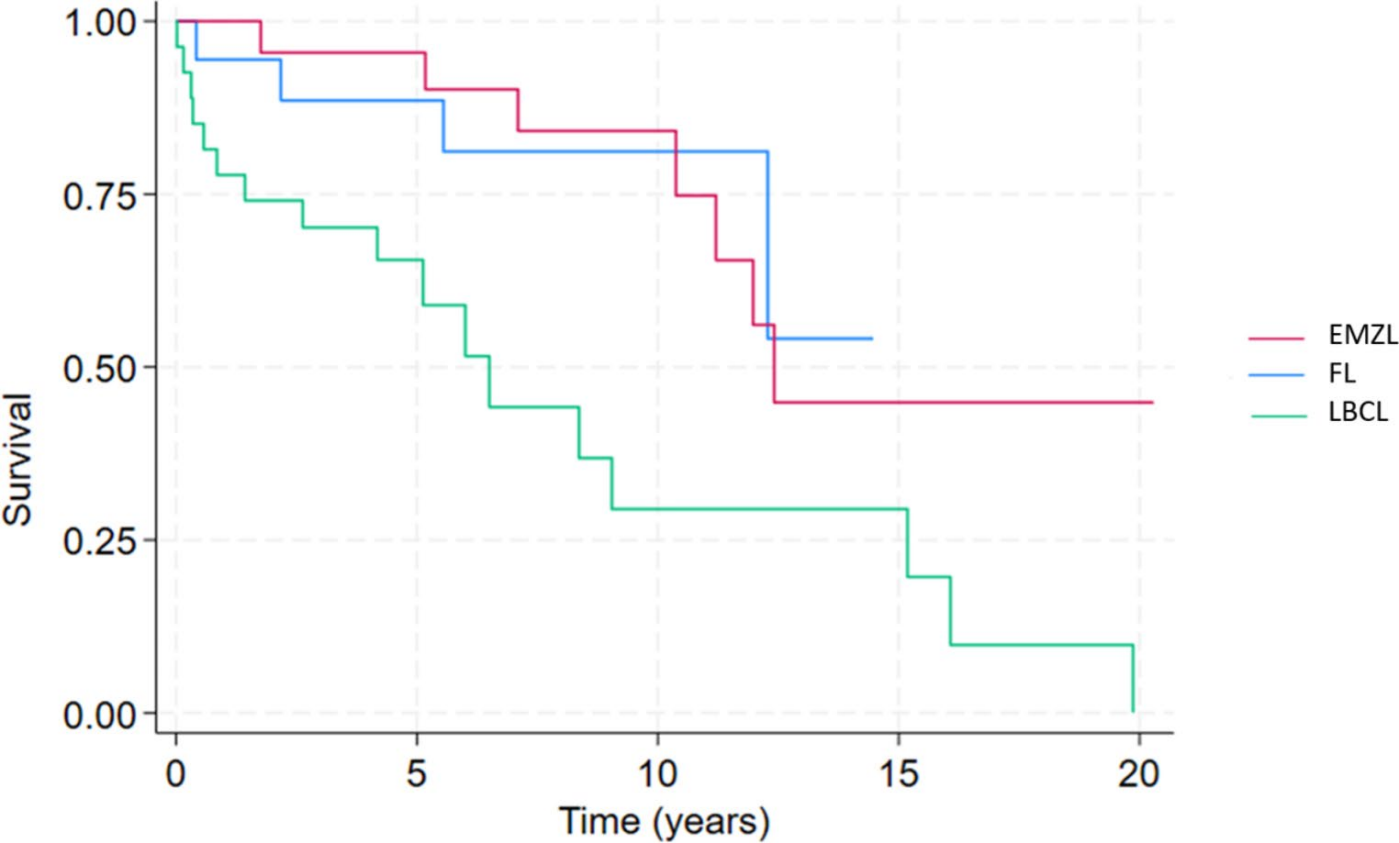
Overall survival of patients with submandibular lymphoma stratified by subtype. FL: Follicular Lymphoma, EMZL: Extranodal Marginal Zone Lymphoma, LBCL: Large B-Cell Lymphoma

**Fig. 4:**
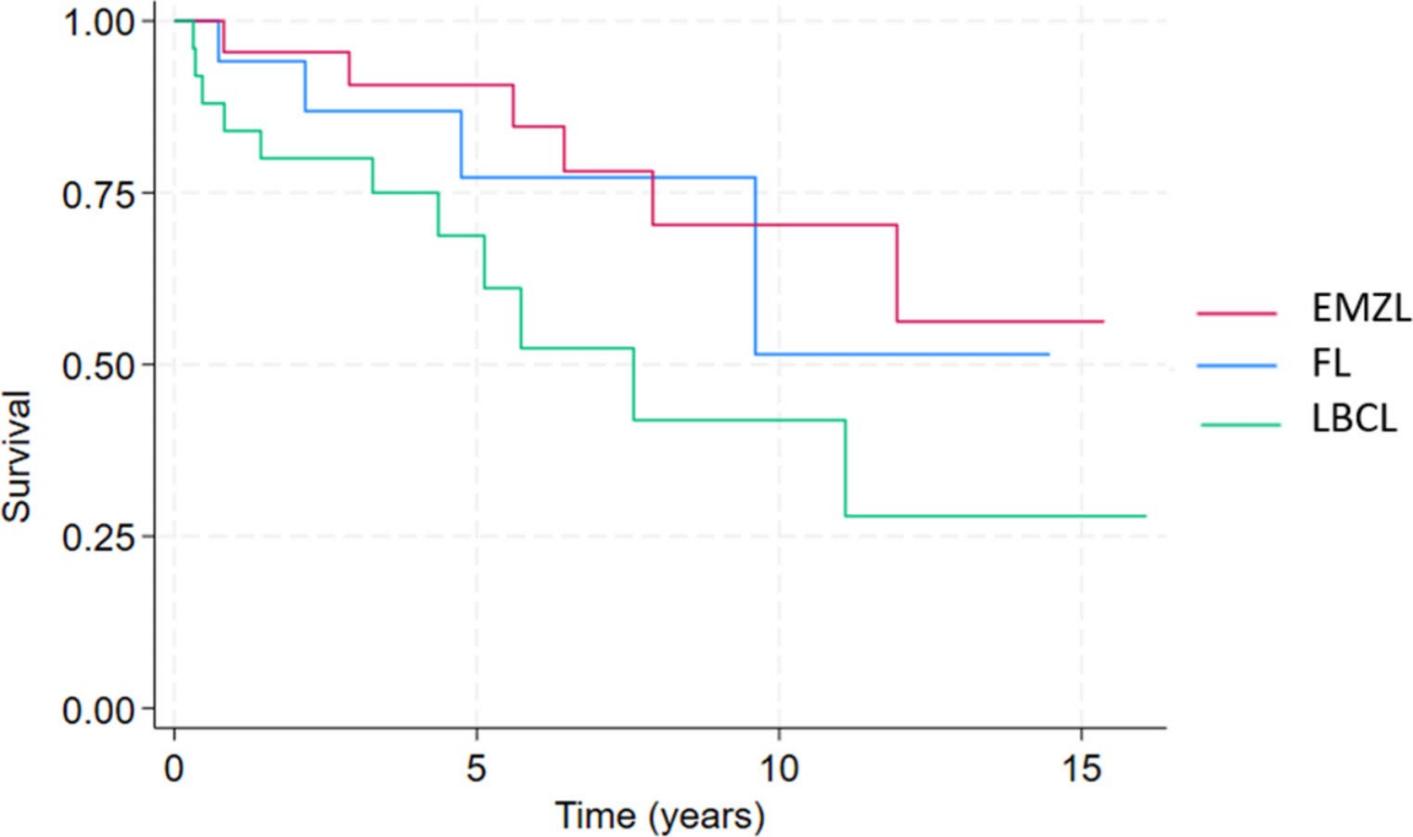
Time to progression in patients with submandibular lymphoma stratified by subtype. FL: Follicular Lymphoma, EMZL: Extranodal Marginal Zone Lymphoma, LBCL: Large B-Cell Lymphoma

The median time to treatment was 25.5 days (*n* = 33) with the most common first-line treatment being chemotherapy (*n* = 25, 75.8%). The response to first-line treatment showed that the majority achieved complete response (*n* = 23, 69.7%). The OS curve indicated a median OS of 6 years (95% CI 4.2-9.0) (Fig. 2). The 5-year OS was 65% and the 10-year OS was 29%. Median TTP was 7.6 years (95% CI 4.4–11.1) (Fig. 3). The logrank test comparing LBCL to other lymphoma types (EMZL and FL) indicated a statistically significant poorer difference in OS (*p* = 0.001) and in TTP (*p* = 0.02) (Fig. 4).

### Features of extranodal marginal zone lymphomas in the submandibular gland

Among the EMZL cases, the majority were female (*n* = 17, 63.0%), with a median age at presentation of 59 years (range 36–85 years). Most EMZL cases were primary lymphomas (*n* = 23, 85.2%) with a predominant unilateral presentation (*n* = 24, 88.9%). Staging according to the Ann Arbor classification revealed that 59.3% of EMZL cases were stage IE with the majority of tumours reported to be between 2 and 5 cm (*n* = 10, 37%) (Table 1).

The most common first-line treatment for EMZL was radiotherapy (*n* = 16, 59.3%) with the response to treatment, according to Lugano criteria, in the majority of cases being complete response (*n* = 14, 60.9%). The OS curve indicated a median OS of 12.4 years (95% CI 6.0-15.2) (Fig. 2). The 5-year OS was 95%, and the 10-year OS was 83%. Median time to progression (TTP) was not reached within the follow-up period (95% CI 7.9–11.9) (Fig. 3). The logrank test comparing EMZL to other lymphoma types (FL and LBCL) indicated a borderline statistically significant difference in OS (*p* = 0.05) and was not significant for TTP (*p* = 0.07). Transformation rate to LBCL was 4.3% (*n* = 1)(Fig. 4).

### Features of follicular lymphomas in the submandibular gland

Of the 29 FL cases there was a notable female predominance (*n* = 19, 65.5%) with the median age at presentation of 68 years. The Ann Arbor staging for FL was more evenly distributed across stages I, II, and III, with fewer cases at stage IV. Common characteristics at presentation can be seen in Table 1.

The median time to treatment was 36 days (*n* = 29) with chemotherapy being the most frequently used first-line treatment (*n* = 11, 37.9%). Following first-line treatment half of the patients (*n* = 15, 51.7%) achieved a complete response. The Kaplan Meier curve showed that the median OS was not reached within the follow-up period (Fig. 2). The 5-year OS was 89%, and the 10-year OS was 81%. Median TTP was also not reached within the follow-up (Fig. 3). The logrank test comparing LBCL to other lymphoma types (EMZL and LBCL) indicated no statistically significant difference in OS (*p* = 0.2) and in TTP (*p* = 0.6). Transformation rate to LBCL was 5% (*n* = 1) (Fig. 4).

### Clinical features of other non-hodgkins lymphomas in the submandibular gland

Among the 12 cases classified as others (11.9%), the distribution included Small Lymphocytic Lymphoma (SLL) (*n* = 4), MCL (*n* = 4), and Peripheral T-cell Lymphoma, Not Otherwise Specified (PTCL-NOS) (*n* = 4). No cases of Hodgkin’s Lymphoma were seen. Ann Arbor staging showed that none of the cases were at stage IE, but a considerable proportion of cases at stage III (*n* = 4, 33.3%) and at stage IV (*n* = 7, 58.3%). Bone marrow involvement was noted in 58.3% of cases (*n* = 7).

### Association with autoimmune disease and COX-regression

There was an increased relative risk of association with autoimmune disease compared to the reference group by approximately 2.7 times, although not statistically significant. (Table 3).

**Table 3:** Relative risk (RR) and 95% confidence intervals (CI) of lymphoma subtypes having autoimmune disease. Subtypes compared to diffuse large B-cell lymphoma

Subtype	RR	Lower CI	Upper CI
DLBCL	1	REF	REF
EMZL	2.67	0.45	16.00
FL	0	0	0
MCL	0	0	0

**Table 4:** Cox Regression Analysis showing Hazard Ratios (HRs) for factors affecting overall survival in patients with lymphoma of the submandibular gland

Cox regression model of overall survival (OS) for patients with lymphoma of the submandibular gland				
Univariate model				
Variable	HR	Lower 95% CI	Upper 95% CI	p
**Female Sex**	0.71	0.34	1.45	0.342
**Age > 60 Years**	3.41	1.38	8.43	0.008
**Stage III-IV**	1.15	0.50	2.64	0.744
**Presence of B Symptoms**	1.43	0.59	3.51	0.430
**Bone Marrow Positive**	0.93	0.41	2.10	0.861
**Elevated LDH**	0.74	0.31	1.77	0.496
**Surgery as Part of Treatment**	1.65	0.67	4.04	0.275
**Time to treatment > 30 Days**	0.67	0.32	1.38	0.274
**Tumor Size > 2 cm**	1.49	0.71	3.16	0.295
Multivariate model				
Variable	HR	Lower 95% CI	Upper 95% CI	p
**Female Sex**	0.95	0.42	2.14	0.898
**Age > 60 Years**	4.30	1.59	11.64	0.004
**Stage III-IV**	1.36	0.51	3.64	0.537
**Presence of B Symptoms**	1.93	0.69	5.38	0.210
**Bone Marrow Positive**	1.09	0.35	3.33	0.886
**Elevated LDH**	0.74	0.27	2.06	0.565
**Surgery as Part of Treatment**	1.31	0.48	3.57	0.592
**Time to treatment > 30 Days**	0.65	0.28	1.50	0.310
**Tumor Size > 2 cm**	1.66	0.67	4.12	0.272

For all patients age above 60 years was significantly associated with poorer OS in both univariate (HR 3.41, 95% CI 1.38–8.43, *p* = 0.008) and multivariate analyses (HR 4.30, 95% CI 1.59–11.64, *p* = 0.004) (Table 4). No other variables reached statistical significance in the multivariate model and the univariate analysis.

## Discussion

This study represents one of the largest investigations of submandibular lymphomas, providing comprehensive data from a nationwide Danish cohort of 101 cases diagnosed between 2000 and 2020. LBCL, FL, and EMZL were identified as the most prevalent subtypes. Our findings align with regional studies but show variance in prevalence rates. Comparing our data with NHL in other geographical regions, we observe notable differences, such as a higher incidence of FL relative to EMZL and LBCL in certain Asian populations, while in Western countries, LBCL is generally more prevalent, with EMZL and FL being less frequent [12–16]. These differences suggest potential factors influencing lymphoma subtype distribution.

Examining other extranodal sites like gastric and lacrimal gland lymphomas highlights unique and shared aspects of lymphoma distribution. The histological and environmental exposures of these glands likely contribute to subtype variations. Our prevalence rates for EMZL and LBCL lie in between those of the same subtypes in gastric and lacrimal gland lymphomas (Gastric LBCL 45–59%, Lacrimal LBCL 10–15% and Gastric EMZL 10–37%, Lacrimal EMZL 37–68%) [17–20]. For follicular lymphoma (FL), the prevalence shows significant variation across different sites, constituting 0.5-2% in gastric lymphomas, 10–19% in lacrimal gland lymphomas, and an even higher 28% in our data [17–20]. 

The histological structure of these glands may contribute to the observed differences [21]. The gastric mucosa, composed of columnar epithelium, differs significantly from the acinar structure of the salivary and lacrimal gland [22–24]. Given the similarity in histological structure between the submandibular and lacrimal gland, we might expect a more similar distribution of lymphoma subtypes compared to the gastric site. This structural variation could influence the development and progression of different lymphoma subtypes.

Moreover, the different environmental exposures of these locations may also play a crucial role in the observed subtype distributions. The submandibular gland, located in the oral cavity, is exposed to substances ingested or inhaled, such as tobacco smoke, alcohol, and medications [25–27]. In contrast, the lacrimal gland, located in the orbit, produces tears and is exposed to air pollutants, allergens, and irritants [28, 29]. The stomach’s shared risk factors with the submandibular gland as part of the digestive system and unique risk factors, such as Helicobacter pylori infection, further highlight the distinct lymphoma subtype distributions across these sites [19, 20, 30]. 

Lymphomas in the submandibular gland, including EMZL, LBCL, and FL, are clinically significant due to their rarity and distinct diagnostic challenges. This study shows that these malignancies often manifest as unilateral asymptomatic swellings, with no B-symptoms, bone marrow involvement or elevated LDH, leading to potential diagnostic delays. Compared to other locations, the symptoms suggest expansive rather than invasive growth due to the absence of symptoms like nerve invasion, trismus, and xerostomia, which can be attributed to the anatomical space in the submandibular region allowing tumour expansion without early compression of structures [31]. Unlike more common conditions, submandibular gland lymphomas can be mistaken for benign conditions or other malignancies, because of their nonspecific clinical presentations and imaging [32]. Additionally, the histopathological diagnosis can be challenging, as salivary glands are not a common site for lymphomas, and the tissue architecture can be distorted by the tumour [33]. This necessitates a high index of suspicion and underscores the importance of comprehensive tissue sampling to achieve a definitive diagnosis. The presence of autoimmune conditions in some lymphoma subtypes, particularly EMZL, adds further complexity to the clinical picture, requiring clinicians to consider autoimmune pathology in their differential diagnosis [12]. 

The connection between autoimmune diseases and the development of lymphomas, particularly in the context of submandibular gland involvement, underscores a complex interaction of immune dysregulation that fosters lymphomagenesis. Autoimmune diseases such as Sjögren’s syndrome, Systemic Lupus Erythematosus, and Rheumatoid Arthritis are well-documented to increase the risk of developing certain lymphomas due to chronic immune stimulation and inflammation, which may facilitate oncogenic processes in susceptible cells [34]. Pathophysiologically, the chronic antigenic stimulation seen in autoimmune conditions is proposed to lead to persistent B-cell receptor signaling, which in turn, promotes the survival and proliferation of autoreactive B-cells [35]. Even though our findings should be cautiously interpreted, the relative risk of 2.67 (CI 95% 0.45-16.0) highlight an enhanced prevalence of EMZL among patients with autoimmune diseases, suggesting that the pathogenic mechanisms linking these conditions may involve unique pathways that are particularly active in the submandibular gland. This relationship might be mediated by local immune responses and the specific microenvironment of the salivary gland, which could provide a niche for lymphoma development under continuous autoimmune stimulation [34]. 

For EMZL and FL, our study shows favourable 5-year OS of 95% and 89%, respectively, with EMZL having a 10-year OS of 83% and FL 81%. These rates are consistent with the prognosis observed in similar extranodal locations, such as the lacrimal and gastric lymphomas, suggesting that lymphomas in the submandibular gland may share similar biological behaviours and treatment responses with these sites [17, 36]. The comparable OS across these locations could indicate that the anatomical site may not significantly impact overall prognosis, emphasizing the importance of subtype-specific factors and treatment approaches. LBCL in the submandibular gland presents a more aggressive clinical course, with a 5-year OS of 65% and a 10-year OS of 29%. This is similar to LBCL in other locations, such as the lacrimal gland, where OS are also lower compared to indolent subtypes like EMZL and FL [17, 36]. The poorer prognosis in LBCL underscores the need for aggressive and timely treatment to improve outcomes. The Cox regression analysis highlights the significant impact of age over 60 years on OS, underscoring age as a critical prognostic factor. Surprisingly, traditional poor prognostic indicators like bone marrow involvement and elevated LDH levels did not significantly impact OS in our cohort [37, 38]. This finding may be specific to submandibular gland lymphomas or related to the small cohort with an additional low frequency of these indicators in our study. Interestingly, delays in diagnosis did not significantly affect OS in our cohort. This observation contrasts with the general assumption in oncology that earlier detection typically leads to better outcomes. The indolent nature of certain lymphoma subtypes, such as EMZL and FL, which exhibit slow progression, may explain why delayed diagnosis does not drastically alter the prognosis. In contrast, timely diagnosis remains crucial for aggressive subtypes like LBCL [33]. 

The study’s limitations include selection bias, data consistency and misclassification risks from integrating multiple sources, and potential residual confounding factors affecting internal validity. The strength in utilizing national registries lies in its extensive coverage and representativeness, potentially enhancing the generalizability of the findings. However, limitations are selection bias due to reliance on NPR, which omits patients whose diagnostic biopsies were taken from sites other than the submandibular gland. Although LyFo was used to counter this bias by identifying patients based on imaging irrespective of biopsy site, its dependency on single-person data entry increases the risk of typing errors. Data consistency and misclassification risks are introduced through the integration of multiple sources, potentially leading to inconsistencies, especially in older cases where data collection protocols were less standardized. Efforts to minimize misclassifications were implemented by having diagnoses validated by two hematopathologists, particularly for ambiguous cases or those lacking modern diagnostic tests such as FISH or PCR for specific lymphoma subtypes like LBCL and EMZL. Internal validity was maintained by stratifying data by lymphoma subtype, sex, age, autoimmune disease occurrence, symptoms, and morphology based on established literature and expert consensus within the research group. However, potential residual confounding by unmeasured factors like socioeconomic status or support networks could affect the study outcomes. The retrospective design also limits control over these confounders, impacting the interpretability of causal inferences. Efforts to mitigate non-differential information bias included collecting similar variables from multiple registries to minimize data gaps. Despite this, variable data consistency across sources and historical changes in data collection practices over the past 40 years may introduce bias, particularly affecting the completeness of older cases.

Future research should focus on the molecular and immunological mechanisms driving these lymphomas and developing targeted therapies for improved management. This study underscores the need for subtype-specific treatment approaches to optimize patient outcomes in patients with as well as without autoimmune diseases.
